# Long noncoding RNAs expression profile and functional networks in rheumatoid arthritis

**DOI:** 10.18632/oncotarget.20036

**Published:** 2017-08-08

**Authors:** Donghua Xu, Ye Jiang, Lu Yang, Xixing Hou, Jihong Wang, Weijun Gu, Xiaodong Wang, Lanyu Liu, Juan Zhang, Hongying Lu

**Affiliations:** ^1^ Department of Rheumatology and Immunology, The Affiliated Hospital of Weifang Medical University, Weifang 261000, China; ^2^ Clinical Medicine College of Weifang Medical University, Weifang 261000, China; ^3^ Department of Laboratory Medicine, The First Affiliated Hospital of Nanjing Medical University, Nanjing 210029, China; ^4^ Department of Gynecology and Obstetrics, Weifang Hospital of Maternal and Child Health, Weifang 261000, Shandong Province, China; ^5^ Department of Rehabilitation, Affiliated Huai’an Hospital of Xuzhou Medical College and Second People's Hospital of Huai’an, Huai’an 223001, China; ^6^ Functional Laboratory, Clinical Medicine College of Weifang Medical University, Weifang 261000, China

**Keywords:** long noncoding RNAs, rheumatoid arthritis, toll like receptor

## Abstract

The modifying effects of long noncoding RNAs (lncRNAs) in rheumatoid arthritis (RA) recently have drawn much attention; however, the underlying mechanisms remain largely unknown. Herein, we aim to investigate the expression profile of lncRNAs in RA and identify promising targets for RA diagnosis and treatment. Microarray screening and real-time PCR of lncRNAs were performed by use of serum samples from 3 RA patients and 3 healthy controls. Significantly differentially expressed lncRNAs were verified in serum samples from 43 RA patients and 40 healthy controls by real-time PCR. We found that there were 73 up-regulated and 61 down-regulated lncRNAs as well as 128 up-regulated and 37 down-regulated mRNAs in serum samples of RA patients. Validation in RA clinical samples indicated 5 of these lncRNAs were significantly up-regulated including RNA143598, RNA143596, HIX0032090, IGHCgamma1, and XLOC_002730. Significant association was observed between these lncRNAs and the disease course, erythrocyte sedimentation rate (ESR), rheumatoid factor (RF) as well as anti-cyclic citrullinated peptide (anti-CCP) antibody. Additionally, 55 of the differentially expressed mRNAs were associated with 41 lncRNAs and were involved in signaling pathways of toll like receptors (TLRs), nuclear factor-kappa B (NF-κB), and cytokine, especially the IRF3/IRF7 mediated signaling transduction. Our study firstly shows the specific profile of lncRNAs in the serum of RA patients and potential signaling pathways involved in RA pathogenesis, which may provide novel targets for the diagnosis and treatment of patients with RA.

## INTRODUCTION

Rheumatoid arthritis (RA) is a common autoimmune inflammatory disease, whereas the pathogenesis of it is not fully elucidated. RA is characteristic of systemic and chronic inflammation, abnormal immune response, and irreversible joint destruction [1, 2]. Some extra-articular manifestations, such as cardiovascular diseases, interstitial lung disease and polyangitis, often lead to poor prognosis of patients with RA [3–5]. Accumulated evidence has suggested that aberrant activation of innate and adaptive immune response plays vital roles in the development and progression of RA. Imbalance of cytokines is involved in RA. Many studies have implicated that a variety of cytokines including TNF-a, IL-1, IL-6, IL-17, IL-18, IL-29, IL-33 are involved in the pathogenesis of RA [6, 7]. Thus, biologics targeting those inflammatory cytokines have been extensively investigated and applied to the treatment of RA, such as TNF-a and IL-6. As biotechnology and bioinformatics grow, research in the expression and regulation of human genes in RA has drawn wide attention. During the past few years, increasing evidence has suggested that the abnormal expression and dysregulation of long noncoding RNAs (lncRNAs) may participate in the pathogenesis of autoimmune diseases including RA [8, 9] .

LncRNAs are RNA transcripts with more than 200 nucleotides in length. They are a new class of regulatory RNAs that are not translated into proteins. LncRNAs are transcription products of RNA polymerase II, which are widely expressed in normal tissues of human body and may be aberrantly and specifically expressed in certain pathological tissues. A number of studies have implicated that lncRNAs can be transported and released into the periphery by circulating exosomes, mediating interacts between cells and their microenvironment [10, 11]. During the last decade, lncRNAs has been strongly suggested to be involved in the development of several kinds of diseases, such as cancer, cardiovascular diseases, and rheumatoid diseases [12–14]. Emerging evidence has revealed that lncRNAs are involved in the regulation of certain biological processes, including chromatin remodeling, gene transcription, and protein transport [15]. It has been well established that lncRNAs regulate the differentiation and activation of T cells, B cells, macrophages, and NK cells, and thus affect autoimmunity and immune-related diseases, such as RA, systemic lupus erythematosus (SLE), primary Sjögren's syndrome (pSS), psoriasis, polymyositis/dermatomyositis (PM/DM) and Crohn's disease (CD) [9, 16, 17]. Therefore, elucidating the role of lncRNAs in RA can help to understand the pathogenesis of RA and provide novel promising targets for the diagnosis, treatment and prognosis estimation of RA.

The aim of this study is to investigate the underlying effects of lncRNAs in RA by microarray screening and bioinformatics analysis. The findings will provide new insights into the pathogenesis of RA and help to identify prospective targets for RA.

## RESULTS

### Aberrantly expressed lncRNAs and mRNAs in RA

**Figure 1:**
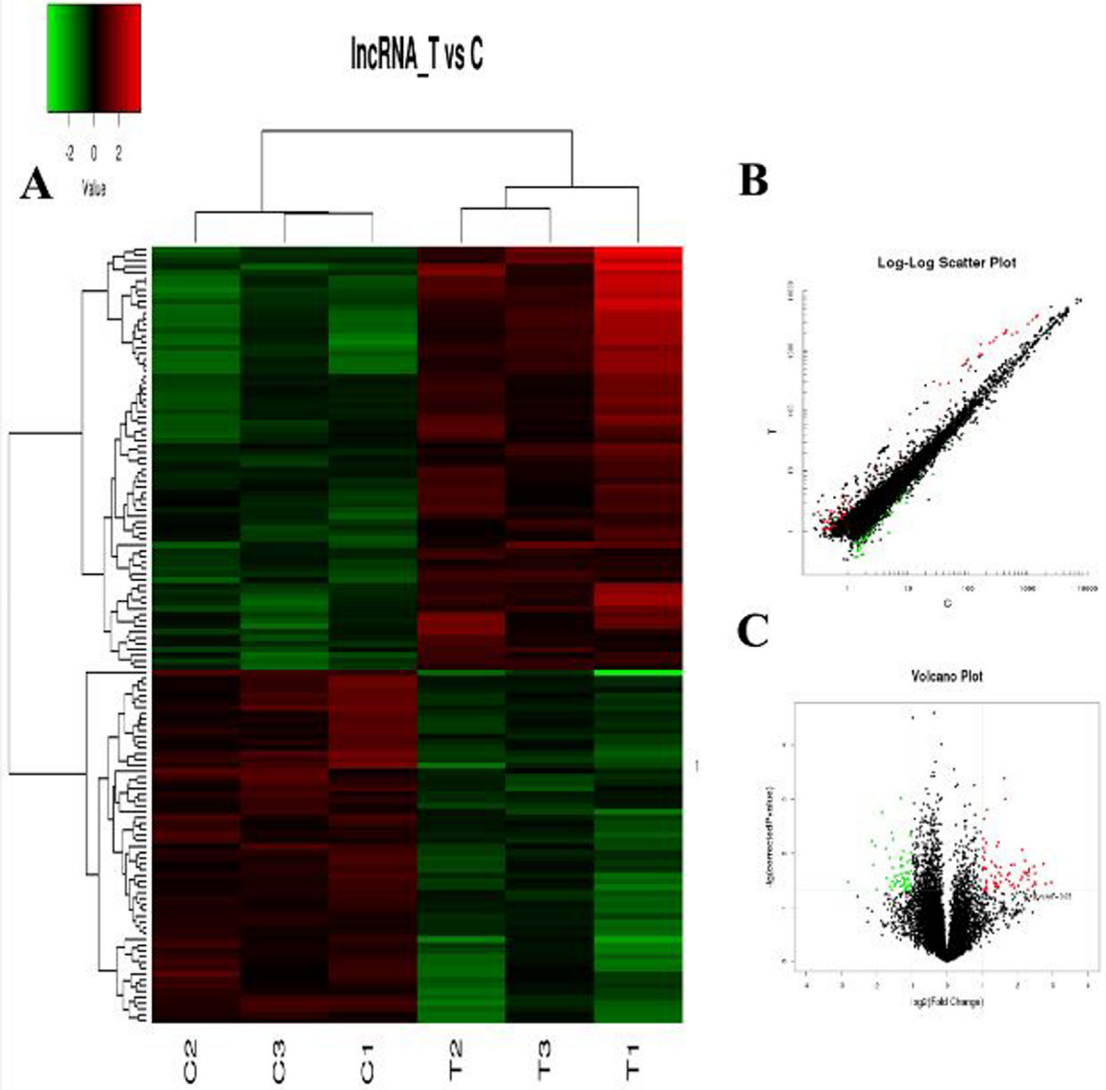
The expression profile of lncRNAs in RA patients compared with healthy controls (**A)** Hierarchical clustering analysis presenting differently expressed lncRNAs. Colors of red and green represent up- and down-regulated genes with changes larger than twofold, respectively. (**B)** Scatter plot showing differently expressed lncRNAs. Red and green plots represent up- and down-regulated genes with changes larger than twofold, respectively. (**C)** Volcano plot showing differently expressed lncRNAs. Red and green plots represent up- and down-regulated genes with changes larger than twofold, respectively.

**Figure 2:**
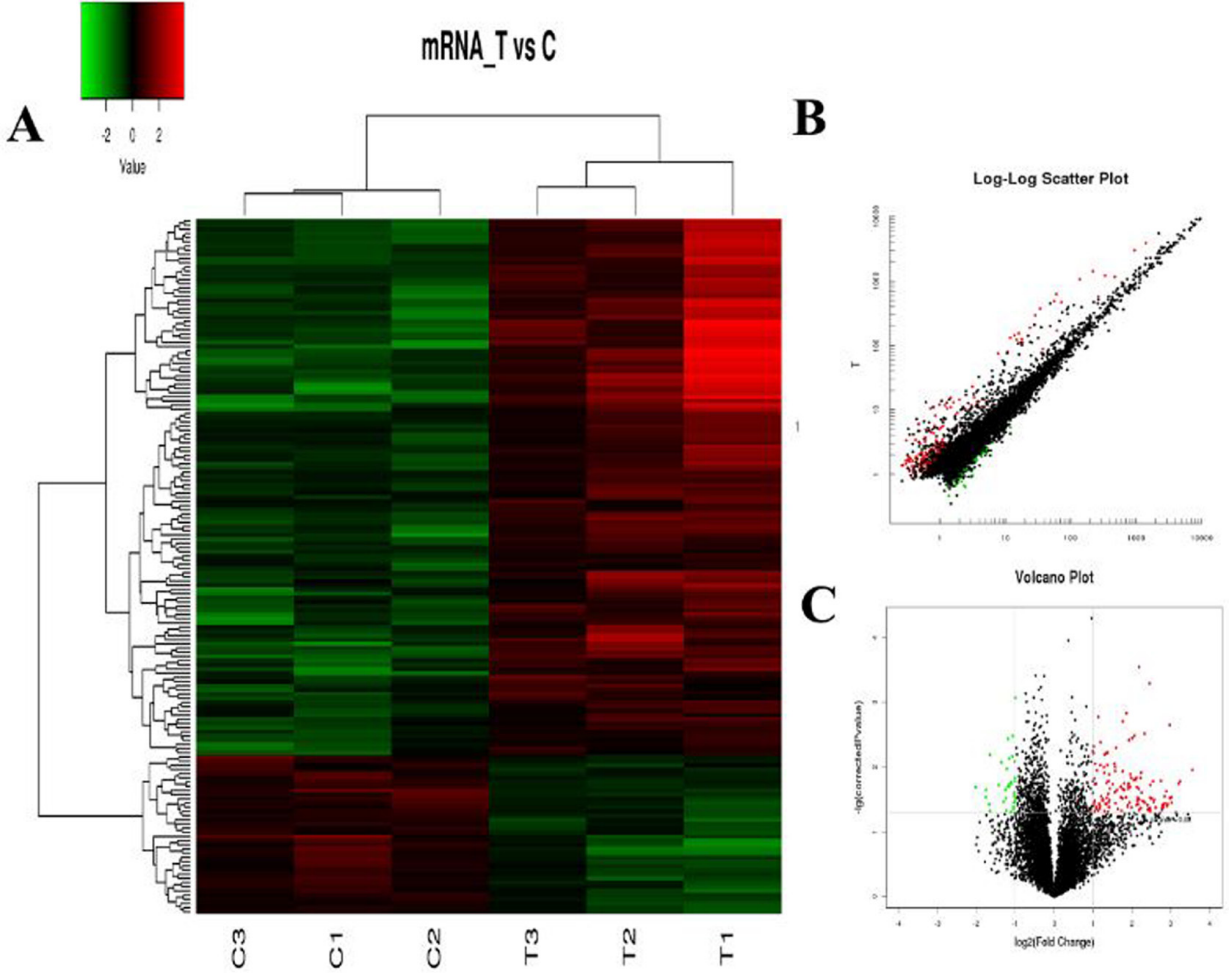
The expression profile of mRNAs in RA patients compared with healthy controls (**A**) Hierarchical clustering analysis presenting differently expressed mRNAs. Colors of red and green represent up- and down-regulated genes with changes larger than twofold, respectively. (**B**) Scatter plot showing differently expressed mRNAs. Red and green plots represent up- and down-regulated genes with changes larger than twofold, respectively. (**C**) Volcano plot showing differently expressed mRNAs. Red and green plots represent up- and down-regulated genes with changes larger than twofold, respectively.

The hierarchical clustering analysis, scatter plot and volcano plot all showed that some lncRNAs and mRNAs were differentially expressed in the serum of RA patients compared with healthy controls (Figure 1A–1C and Figure 2A–2C). A total of 73 up-regulated and 61 down-regulated lncRNAs as well as 128 up-regulated and 37 down-regulated mRNAs were identified in the serum of patients with RA after the microarray screening. Table 1 and Table 2 presented the top 30 aberrantly expressed lncRNAs and mRNAs in RA, respectively.

**Table 1:** Top 30 aberrantly expressed lncRNAs in RA patients compared with healthy controls

LncRNA	*P* value	Fold changes
**Up-regulated**		
RNA147405|p0509_imsncRNA82	0.034815	7.772733
ENST00000561134.1	0.037058	6.968304
HIT000064601	0.015806	6.695212
p44304_v4	0.038641	5.674733
RNA143598	0.023631	5.673976
XR_243720.2	0.034541	5.649367
XR_429995.1	0.034812	5.621383
ENST00000579527.1	0.01753	5.498751
p28385	0.040602	5.334821
TCONS_00007197	0.019452	5.043555
RNA143577	0.021894	5.00845
RNA143578	0.030799	4.922104
RNA143580	0.025897	4.919801
RNA147303|p0407_imsncRNA822	0.047544	4.911092
RNA143589	0.023075	4.780086
RNA143581	0.023414	4.638907
HIX0032090	0.013736	4.616553
RNA143595	0.022496	4.565024
RNA143579	0.027482	4.386317
LIT3528	0.00859	4.378436
RNA143540|tRNA_457_68	0.031161	4.270831
RNA143596	0.040198	4.074681
ENST00000548571.1	0.043473	3.740273
XR_108761.3	0.016348	3.731026
LIT3526	0.037608	3.678755
ENST00000553426.1	0.016396	3.518957
HIT000092395_03	0.039359	3.492784
ENST00000441075.1	0.023534	3.432525
IGHCgamma1	0.025053	3.284312
XLOC_002730	0.030258	3.222785
**Down-regulated**		
p26558	0.034042	6.986983
ENST00000541797.1	0.005979	4.39359
HIT000296997	0.016428	4.269773
RNA33664|snoRNA_scaRNA_260_79	0.007309	4.121303
TCONS_00022112	0.046352	3.983995
ENST00000495580.1	0.001753	3.581599
ENST00000439406.1	0.008247	3.336626
RNA95721|RNS_803_121	0.028929	3.264407
XR_242051.1	0.012273	3.085937
TCONS_00027142	0.041577	3.062286
ENST00000606966.1	0.004208	2.963269
ENST00000525331.1	0.047289	2.935378
ENST00000606879.1	0.018672	2.90775
ENST00000596887.1	0.033564	2.898185
ENST00000439633.1	0.005507	2.886541
TCONS_00009147	0.026974	2.801348
ENST00000422944.1	0.040728	2.796731
ENST00000550263.1	0.036059	2.751807
TCONS_00021834	0.037043	2.744497
ENST00000586610.1	0.032215	2.668683
TCONS_00027516	0.032387	2.652956
ENST00000436429.1	0.025568	2.635222
TCONS_00021014	0.033183	2.536658
ENST00000525429.1	0.041101	2.507953
ENST00000450990.1	0.01297	2.496252
ENST00000563230.1	0.017314	2.493657
TCONS_00028421	0.00096	2.467011
XR_109933.1	0.012023	2.44694
ENST00000604448.1	0.024131	2.442996
nc-HOXC11-108	0.039676	2.400215

**Table 2:** Top 30 differentially expressed mRNAs in RA patients compared with healthy controls

mRNA	*P* value	Fold changes
**Up-regulated**		
CST5	0.011015	11.7354
MYL5	0.016609	9.437377
MTRNR2L2	0.017951	9.170133
ABLIM3	0.035346	8.106463
ATP5A1	0.028899	8.078051
CDK2AP1	0.028824	8.064926
TRAPPC1	0.030183	7.857362
HLA-A	0.002232	7.815082
MAP1LC3B	0.036902	7.535449
MNDA	0.039922	7.465274
CD9	0.02348	7.367939
GABARAPL2	0.028315	7.146566
ATP6V0C	0.036303	7.128572
FCGR2A	0.040717	7.009223
OSBPL9	0.038812	6.904492
RAB11B	0.042927	6.857076
MTRNR2L9	0.045445	6.854809
ND4L	0.016292	6.695147
DUSP6	0.042244	6.100676
TLN1	0.031514	5.967558
GNG10	0.034438	5.903362
MLH3	0.016582	5.829806
CDC14B	0.030726	5.664988
LOC100130865	0.04633	5.565929
PTPN22	0.018824	5.555936
A_21_P0013791	0.029625	5.535323
FURIN	0.048091	5.514076
TCP11L2	0.042912	5.49967
USP17L25	0.000509	5.468273
ND4	0.044486	5.341476
**Down-regulated**		
ACACA	0.020405	4.04911
RAB6C-AS1	0.028936	3.383973
DNAH2	0.022414	3.373326
GGCT	0.037562	3.187807
TRIP10	0.006481	3.142754
CSF1R	0.047671	3.142102
TMEM229B	0.018878	2.677905
FILIP1L	0.008344	2.559852
A_33_P3244828	0.034094	2.470736
lnc-GRIK1-AS2-1	0.022228	2.376733
BCL6	0.030188	2.364881
INPP5F	0.046803	2.35886
NUTM2B	0.010629	2.329807
CYP2R1	0.019688	2.279476
NELL1	0.003652	2.272997
A_33_P3398005	0.00733	2.235301
HOXD10	0.019771	2.223335
A_33_P3333364	0.01795	2.191913
ZNF358	0.017607	2.185415
A_33_P3290301	0.017789	2.182656
POU4F3	0.04991	2.171979
lnc-CIB4-1	0.017052	2.145348
TMCO2	0.04851	2.144128
SSX3	0.017698	2.13033
A_33_P3287058	0.006785	2.109284
lnc-RASA1-5	0.003285	2.092431
ADCYAP1R1	0.043844	2.063525
SP140	0.041264	2.060501
lnc-TEX261-2	0.034831	2.046709
MGAT4D	0.028391	2.041796

### LncRNA expression profile in the serum of RA

**Figure 3:**
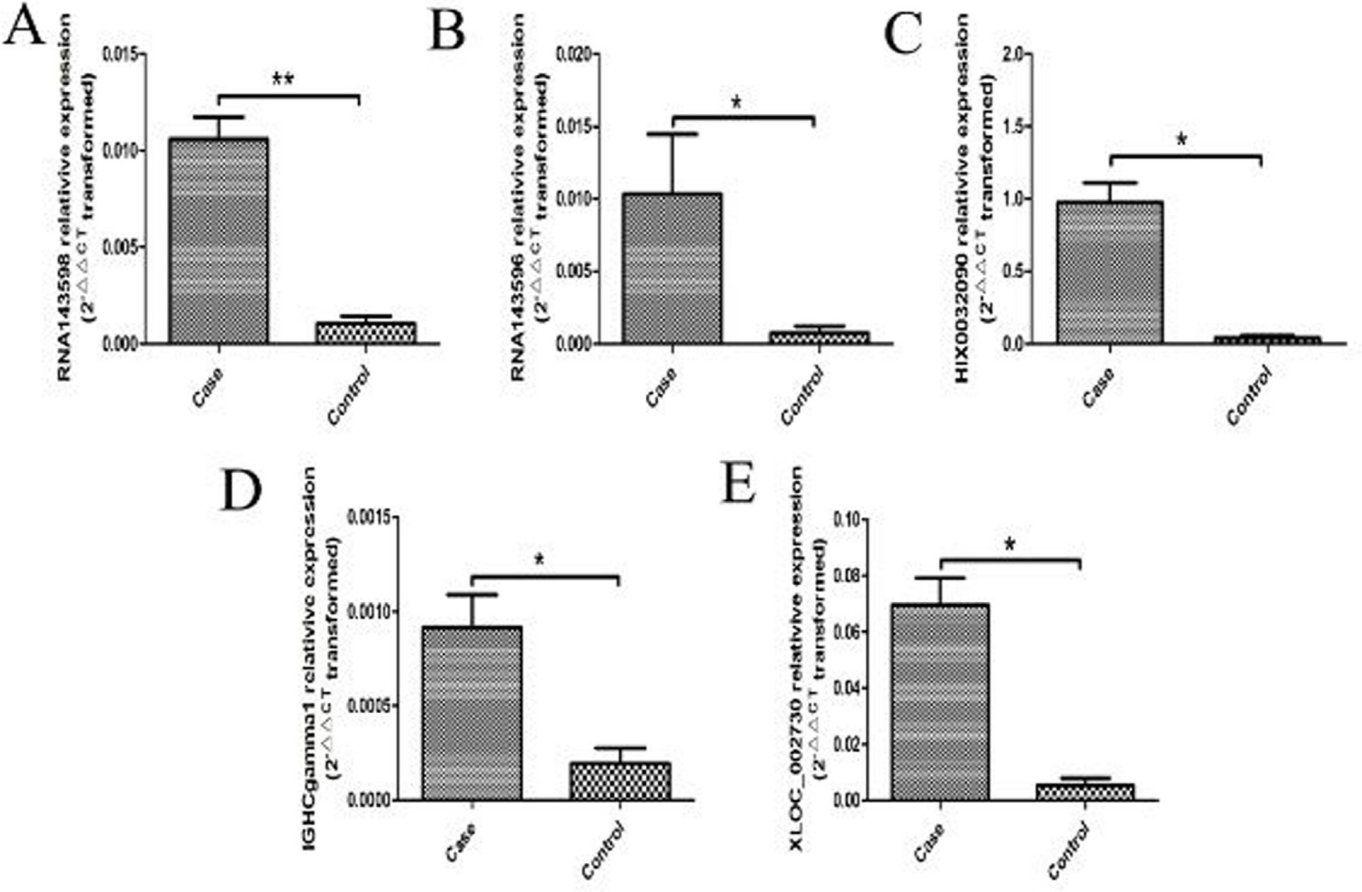
5 aberrantly expressed lncRNAs validated by real-time PCR in the serum from 43 RA patients and 40 healthy controls (**A**) RNA143598 was significantly increased in RA compared with healthy controls (**P < 0.01). (**B**) RNA143596 was significantly increased in RA compared with healthy controls (*P < 0.05). (**C**) HIX0032090 was significantly increased in RA compared with healthy controls (*P < 0.05). (**D**) IGHCgamma1 was significantly increased in RA compared with healthy controls (*P < 0.05). (**E**) XLOC_002730 was significantly increased in RA compared with healthy controls (*P < 0.05).

Most of the aberrantly expressed lncRNAs were validated in the serum samples from 43 RA patients and 40 healthy controls by real-time PCR. 5 of these lncRNAs were found to be significantly up-regulated in the serum of RA patients when validating by real-time PCR, including RNA143598, RNA143596, HIX0032090, IGHCgamma1, and XLOC_002730 (Figure 3).

### Association between differentially expressed lncRNAs and clinical characteristics of RA

**Figure 4:**
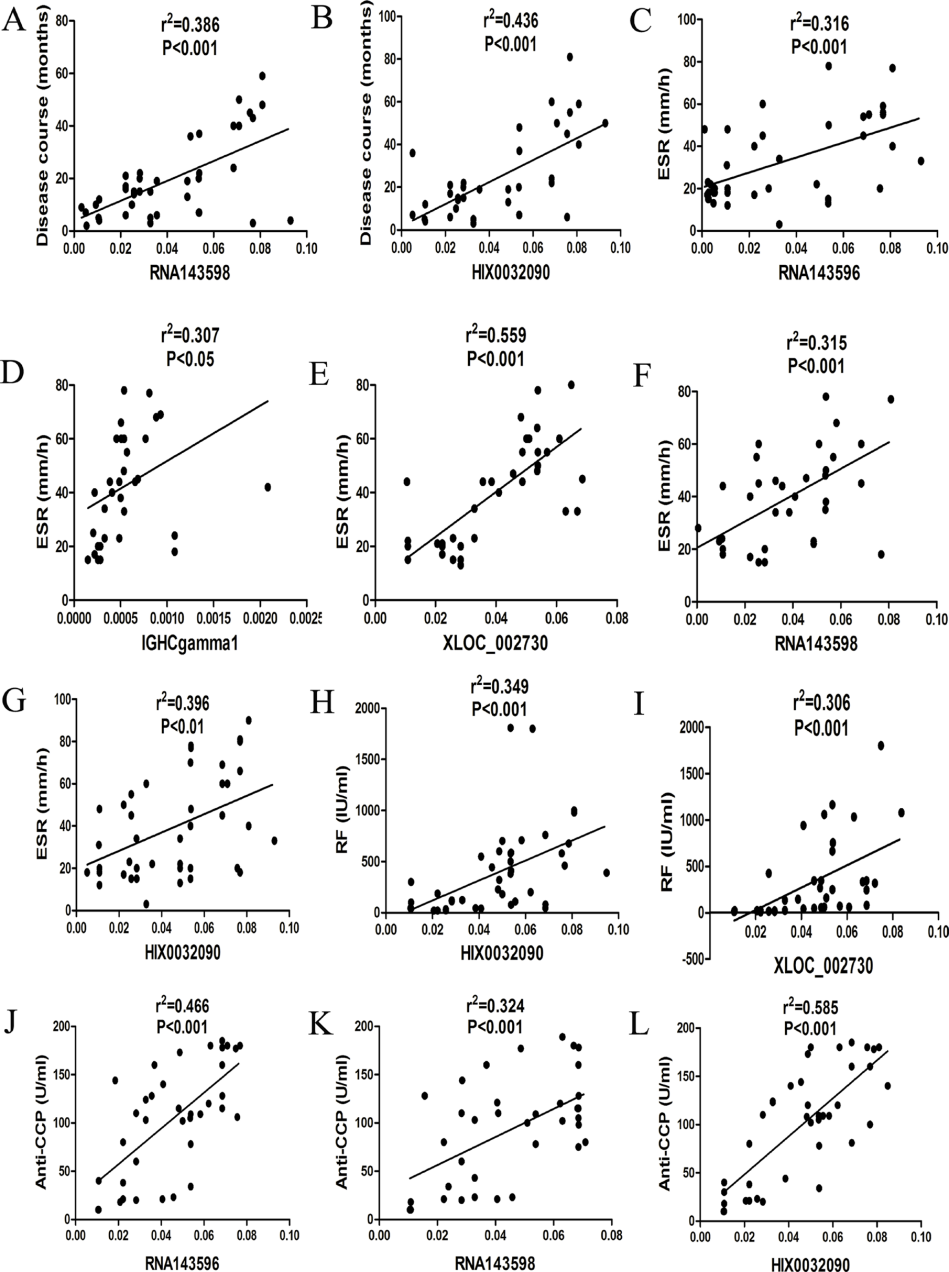
Correlations between aberrantly expressed lncRNAs and clinical characteristics of RA (**A**) RNA143598 was significantly related to the disease course of RA. (**B**) HIX0032090 was significantly related to the disease course of RA. (**C**) RNA143596 was significantly related to ESR in RA. (**D**) IGHCgamma1 was significantly related to ESR in RA. (**E**) XLOC_002730 was significantly related to ESR in RA. (**F**) RNA143598 was significantly related to ESR in RA. (**G**) HIX0032090 was significantly related to ESR in RA. (**H**) HIX0032090 was significantly related to RF in RA. (**I**) XLOC_002730 was significantly related to RF in RA. (**J**) RNA143596 was significantly related to anti-CCP Ab in RA. K RNA143598 was significantly related to anti-CCP Ab in RA. (**L**) HIX0032090 was significantly related to anti-CCP Ab in RA.

Significant association was observed between these up-regulated lncRNAs and the disease course, erythrocyte sedimentation rate (ESR), rheumatoid factor (RF), anti-cyclic citrullinated peptide antibody (anti-CCP Ab) of RA (Figure 4).

### Functional prediction of aberrantly expressed mRNAs

**Figure 5:**
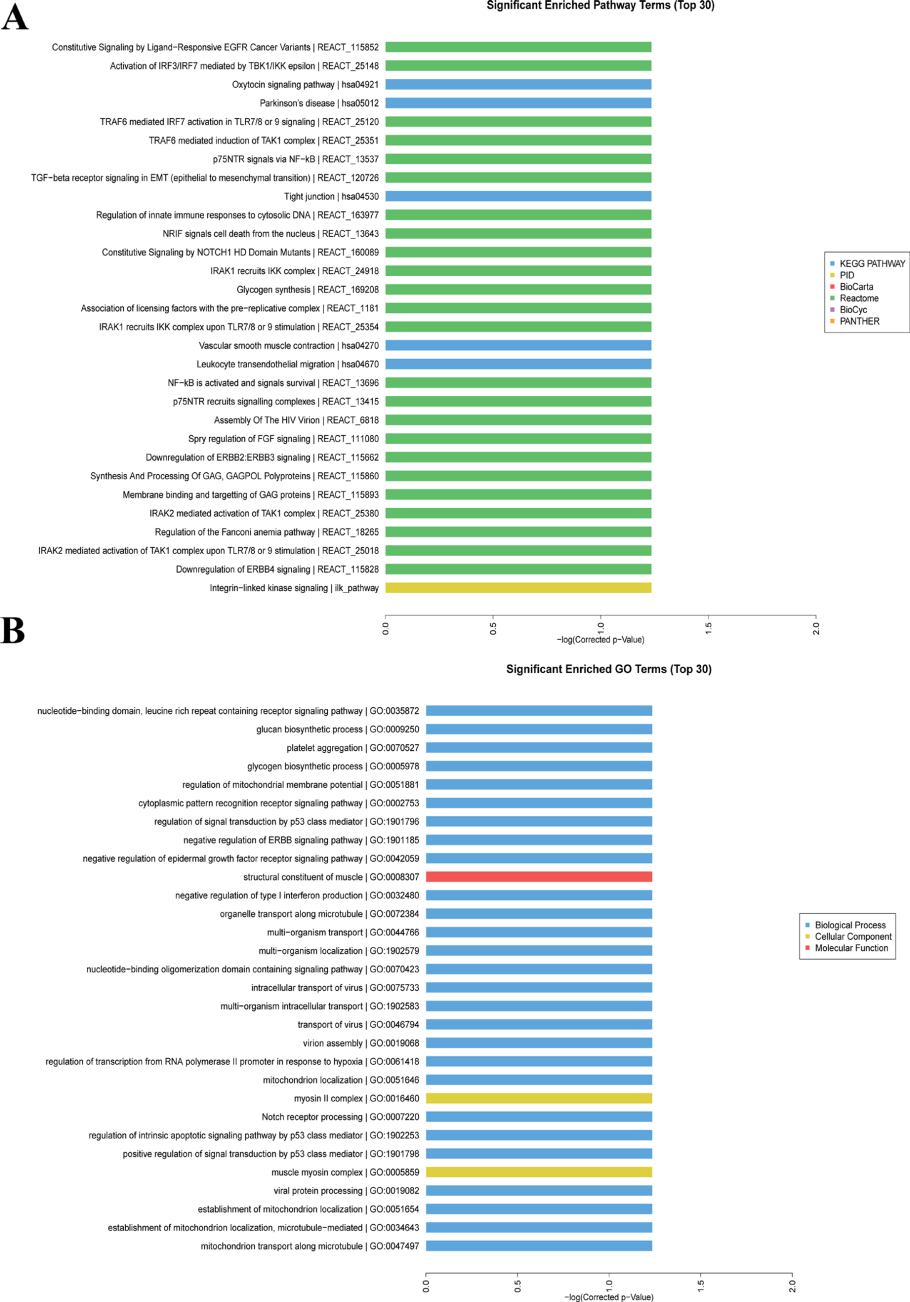
GO and pathway enriched analyses for differently expressed mRNAs (**A**) Significantly enriched pathway terms of differently expressed mRNAs.(**B**) Significantly enriched GO terms of differently expressed mRNAs.

Figure 5A showed the top 30 significant enriched pathway terms with regard to the aberrantly expressed mRNAs in RA, primarily including toll like receptors (TLRs), nuclear factor-kappa B (NF-κB), and cytokine signaling pathways. Besides, the interferon regulatory factor (IRF3/IRF7) mediated signaling transduction was dominant in those pathways. The top 30 significant enriched GO terms were presented in Figure 5B, which were mainly involved in the biological processes, cellular components, and molecular functions of aberrantly expressed mRNAs in RA.

### Association between lncRNAs and mRNAs

**Figure 6:**
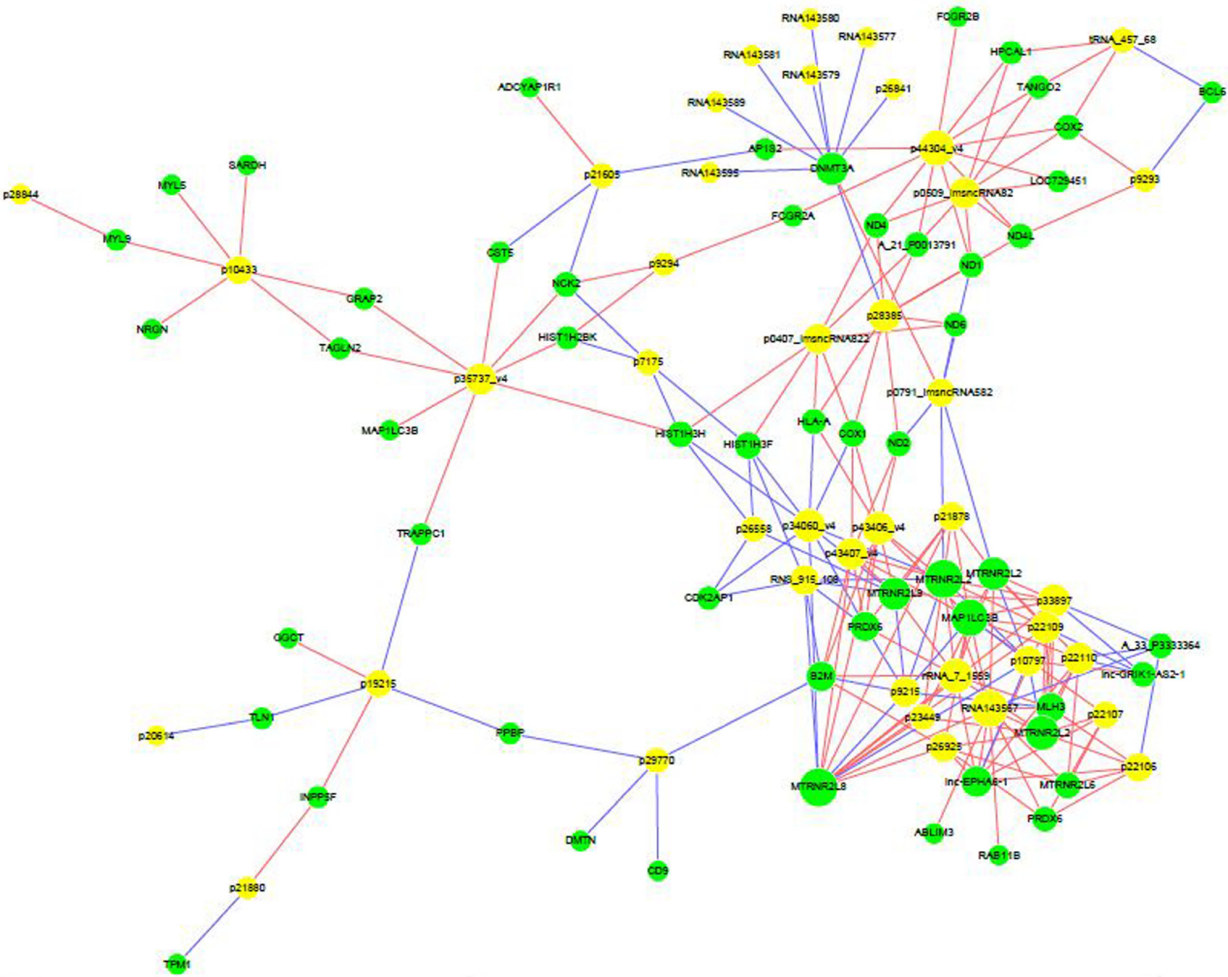
Correlation between lncRNAs and mRNAs LncRNA-mRNA network graph showed that 55 differentially expressed mRNAs were associated with 41 lncRNAs.

Computational analysis revealed that 55 mRNAs were associated with 41 differentially expressed lncRNAs (Figure 6). These significantly associated lncRNAs-mRNAs pairs were primarily involved in signaling pathways of TLRs, NF-κB, and cytokine, which might contribute to the pathogenesis of RA and influence the prognosis of RA patients.

## DISCUSSION

LncRNAs, as a class of noncoding RNAs, play critical roles in the regulation of autoimmunity and maintenance of homeostasis [18, 19]. Multiple lines of evidence have suggested that the dysregulation of lncRNAs is involved in a variety of human diseases, including metabolic disease, cancer and rheumatoid disease [19–21]. A spectrum of mechanisms has been elucidated, such as the control of mRNA decay, recruitment of epigenetic modifier proteins, and regulation of microRNAs expression [21–23]; however, mechanisms of lncRNAs-mediated regulation in RA remain not fully understood. Understanding of lncRNAs-mediated regulation is essential for investigating prospective and novel targets for the diagnosis, treatment and prognosis estimation of RA.

RA is one of the most frequent rheumatoid diseases worldwide with kinds of strong autoimmune components, such as autoantigens of citrullinated proteins [2, 3]. To the best of our knowledge, inflammation is closely related to the occurrence of RA, disease activity and prognosis. Long sustained and chronic autoimmune inflammation in the synovium can lead to destruction of joints and deformity. LncRNAs are involved in the expression of inflammatory related genes, epigenetics, inflammatory signal transduction and other biological processes by acting as guidance molecules, signal molecules, decoy molecules, and cytoskeleton molecules [24]. Therefore, it can be concluded that lncRNAs may confer modifying effects on the development and progression of RA. Recent progress has suggested that the role of lncRNAs in RA could be far more prevalent than previously appreciated. LncRNA ANRIL is the first documented lncRNA involved in the pathogenesis of RA [25]. Additionally, lncRNA ANRIL can influence inflammation primarily by modulating NF-κB and its downstream signaling pathway, and thus participates in biological processes of glucose metabolism and inflammatory response and ultimately causes coronary heart disease, vasculitis and other inflammation-related diseases [25, 26]. Aterido A et al. have found that lncRNA FAM66C was abnormally expressed in CD4+ T lymphocytes and could affect CD4+ T cells-mediated immune response in RA [27]. Increased expression of lncRNA Hotair can induce the migration of more macrophages to inflammatory sites and the amplification of local inflammatory response, which ultimately promotes the progression of RA [28]. Taken together, lncRNAs-mediated inflammatory response and immune regulation play crucial roles in the development and progression of RA. Nonetheless, little is known about the molecular mechanism in RA pathogenesis regarding lncRNAs-mediated modulation, which warrants further investigation in more future studies.

In the present study, we tried to find the differentially expressed lncRNAs and mRNAs profiles in the serum of RA patients by microarray screening. 5 significantly differentially expressed lncRNAs were identified in serum samples from patients with RA, including RNA143598, RNA143596, HIX0032090, IGHCgamma1, and XLOC_002730. Besides, significantly positive association was observed between these lncRNAs and the disease course, ESR, RF and anti-CCP antibody of patients with RA. The correlation analysis showed that RNA143598 and HIX0032090 were significantly associated with the disease course and ESR level in patients with RA. In addition, increased levels of RF were observed in RA patients with XLOC_002730 and HIX0032090 up-regulated in the serum among RA patients. Moreover, the expression of RNA143596, RNA143598 and HIX0032090 was also positively related to the level of anti-CCP antibody in the serum. It has been well established that elevated level of RF is highly associated with synovitis, vasculitis and extra articular symptoms, while increased level of anti-CCP antibody is closely related to bone erosion and destruction [29]. Accordingly, all these findings have suggested important values of lncRNAs in the diagnosis and prognosis adjustment of RA. The differentially expressed lncRNAs are associated with inflammatory response and autoimmunity, and thus influence RA occurrence, progression and prognosis.

As shown in the pathway enrichment analysis, TLRs/NF-κB signal pathways are highly enriched in RA associated with the aberrantly expressed mRNAs. Thus, TLRs/NF-κB mediated inflammation may contribute to the development of RA. Currently published studies have suggested a crucial role of TLRs/NF-κB signaling transduction in the development of some autoimmune diseases including RA, Sjogren's syndrome and systemic sclerosis [30–32], supporting multiple promising therapeutic targets for these diseases. Similarly, we have previously found that LPS/TLR4/NF-κB signaling pathway contributed to the pathogenesis of RA [7]. Taken together, LPS/TLR4-mediated inflammation is involved in RA dependent on NF-κB signaling activation. Nonetheless, except for TLR4, other common types of TLRs, such as TLR3, TLR7, and TLR9, may also contribute to the RA pathogenesis, which is needed to be further investigated in more future studies, particularly regarding the underlying molecular mechanisms of lncRNAs involved in RA pathogenesis.

In this study, the cytokine signaling pathway has been significantly enriched by the bioinformatics analysis in the present study, especially the IRF3/IRF7 mediated signaling transduction. IRF-3 and IRF-7 are the vital transcriptional factors for the generation of IL-28A and IL-28B, whereas IRF3 and NF-κB are critical transcript factors for the production of the IL-29 [33]. IL-29, an important molecule of type III interferon family, has been demonstrated to be associated with enhanced inflammation in the development of RA in our previous study [7]. Type III interferon mainly consists of IL-29, IL-28A and IL-28B. In addition, several published studies have implicated that IL-29 could affect the inflammatory response involved in the pathogenesis of certain diseases by activation of IRFs signaling pathways [33, 34]. Moreover, the study by Xu et al. has revealed that IL-29 might enhance TLRs-mediated production of inflammatory cytokines in synovial fibroblasts [30], suggesting a crucial role of IL-29/TLRs signaling pathways in RA pathogenesis. However, the potential effects of type III interferon family and IRFs related signaling transduction in RA remain largely unknown. We hypothesize that IL-29/IRFs signaling pathways might participate in the development and progression of RA. However, we fail to elucidate the underlying effects of lncRNAs/IL-29/IRFs in RA by use of cell and/or animal models in this study. More future studies are warranted to demonstrate the precise regulatory mechanisms of lncRNAs/IL-29/IRFs signal involved in RA.

In summary, this study, for the first time, shows specific profiles of lncRNAs and mRNA in the serum of RA patients and potential lncRNA-mRNA networks involved in RA. There are a total of 73 up-regulated and 61 down-regulated lncRNAs as well as 128 up-regulated and 37 down-regulated mRNAs in RA. LncRNAs of RNA143598, RNA143596, HIX0032090, IGHCgamma1, and XLOC_002730 are significantly up-regulated in the serum of RA patients. The differentially expressed lncRNAs are closely associated with the inflammatory response and autoimmunity. Findings in this study will support novel promising targets for RA.

## MATERIALS AND METHODS

### Study subjects and sample preparation

43 RA patients and 40 healthy controls were recruited from the affiliated hospital of Weifang Medical University between September, 2015 and March, 2016. Controls were from the same hospital for health examination. All fresh blood samples were separately collected and sequentially centrifuged at 2000 rpm for 10 min. 1ml cell-free serum from the supernatant was sucked out and stored at -80°C for further detection. Characteristics of all patients and controls were summarized in Table 3.

**Table 3:** Characteristics of RA patients and controls

	Patients (*n* = 43)	Controls (*n* = 40)
Age (mean ± SD)	49.2 ± 19.0	47.8 ± 17.2
Sex (Women/Man)	30/13	25/15
Disease course (year)	2.5 ± 1.1	-
Score of disease activity in 28 joints	3.8 ± 1.0	-
ESR (mm/h)	57.1 ± 13.7	11.2 ± 4.1
CRP (mg/L)	34.1 ± 5.2	5.1 ± 1.2
RF (IU/ml)	116.1 ± 23.4	9.7 ±1.0
Anti-CCP (U/ml)	71.5± 19.4	30.9 ± 14.2

### RNA isolation and real-time PCR

Total RNAs were isolated from serum samples by use of Plasma/Serum RNA Purification Mini Kit (Norgen Biotek Corp., Thorold, Canada) according to the manufacturers’ protocols. Purified total RNAs were quantified using a NanoDrop 1000 (Thermo Fisher Scientific, Waltham, MA, USA). Then, cDNAs were synthesized from 0.5 μg RNAs for further assay based on the instructions of PrimeScript™ RT reagent Kit (Takara, Tianjin, China). Real-time PCR was carried out in triplicate assay in accordance with the specifications of SYBR Green Mastermix kit (Takara, Tianjin, China). A total of 5 ng cDNA template was used for real-time PCR assay. Random primers were used in experiments. The expression of each lncRNA was represented as fold changes using the 2^-ΔΔCT^method and normalized to housekeeping gene GAPDH. Primer sequences used in validation of lncRNAs in this paper were listed in Table 4.

**Table 4:** primer sequences used in validation of lncRNAs

LncRNA	Up-stream primer sequence (5′ to 3′)	Down-stream primer sequence (5′ to 3′)
RNA143598	TTACACAAGCAAGCATCGCC	TATCACTGCTGTCTCCCGTG
RNA143596	CAAAACACTTTGCTCGGCCA	TAATCGTATGGCTGCGGTGG
HIX0032090	ACTGCTCGCCAGAACACTAC	GGTGAGGTTGATCGGGGTTT
IGHCgamma1	GTGACGGTGTCGTGGAACTC	GTGTTGCTGGGCTTGTGATT
XLOC_002730	TTGCTATGTTATGCCCGCCT	CGGTACCCTAACCGTGCAAA

### Microarray screening

Capitalbio Agilent LncRNA + mRNA Human Gene Expression Microarray V4.0 was used to screen differentially expressed lncRNAs and mRNAs in the serum of RA patients (Capitalbio Corp., Beijing, China). In this study, serum samples from 3 patients and 3 controls were randomly selected for microarray analysis. It was considered to be statistically significant between the two groups when the fold changes for differentially expressed lncRNAs and mRNAs were larger than 2.0 and the *P* value for *t*-test was less than 0.05. Most of the differentially expressed genes were validated by real-time PCR, especially those lncRNAs which co-expressed with mRNAs.

### Bioinformatics analysis

After microarray screening for lncRNAs and mRNAs in the serum samples from RA patients and controls, the hierarchy clustering, gene ontology (GO) functional enrichment and pathway enrichment analyses were then performed for further estimation by use of KOBAS (KEGG Orthology Based Annotation System) software. In order to predict the potential regulatory effects of differentially expressed lncRNAs on mRNAs, an lncRNA-mRNA co-expression network was conducted by bioinformatics analysis.

### Statistical analysis

Data were shown as mean ± SEM. We used independent-Samples T test or One-Way ANOVA for statistical analysis. A two-sided *P* < 0.05 was regarded to be statistically significant. Softwares of SPSS (version 16.0) and Graphpad (version 5.0) were applied for statistical analysis.
